# Dual Toroidal Dipole Resonance Metamaterials under a Terahertz Domain

**DOI:** 10.3390/ma11102036

**Published:** 2018-10-19

**Authors:** Shuang Wang, Song Wang, Quan Li, Xiaoli Zhao, Jianyu Zhu

**Affiliations:** 1 School of Electronic Engineering, Tianjin University of Technology and Education, Tianjin 300222, China; 13752793692@163.com (S.W.); liquanstudy@126.com (Q.L.); 15202237042@139.com (X.Z.); ziyuyu2050@163.com (J.Z.); 2 National-Local Joint Engineering Laboratory of Intelligent Manufacturing Oriented Automobile Die & Mould, Tianjin University of Technology and Education, Tianjin 300222, China

**Keywords:** metamaterials, terahertz, toroidal dipole

## Abstract

We proposed and fabricated a flexible, planar, U-shape-modified structure metamaterial (MM) that was composed of two metallic pattern layers separated by a polyimide layer, where each metallic pattern layer consists of two U-shaped split ring resonators (USRRs). The coupling effect between the two USRRs in the same metallic layer was vital to the formation of dual toroidal dipole (TD) resonances. The measured and simulated results showed that both low quality factor (Q) (~1.82) and high Q (~10.31) TD resonances were acquired synchronously at two different frequencies in the MMs by adjusting the distance between the two coplanar USRRs. With the interaction of the USRRs, the energy levels of the USRRs were split into inductance-capacitance (LC)-induced TD resonance at low frequency and dipole-induced TD resonance at high frequency. Thus, the electric multipole interaction played an important role in determining the energy level of the TD resonance. The better strength of the high frequency TD resonance can be confined to an electromagnetic field inside a smaller circular region, and thus, a higher Q was obtained. In order to investigate the TD mechanism more in depth, the power of the electric dipole, magnetic dipole, electric circular dipole, and TD were quantitatively calculated. Dual TD MMs on a freestanding substrate will have potential applications in functional terahertz devices for practical applications.

## 1. Introduction

Toroidal resonance was discovered by Zel’dovich in 1957 [1], and can enhance field confinement by concentrating a magnetic field in a small circular region. Since then, the interactions between toroidal resonance and external electromagnetic fields have become a subject of growing interest [2,3,4,5,6,7,8,9,10,11,12,13,14]. As the simplest resonance of a toroidal multipole, toroidal dipole (TD) resonance is created by currents flowing on the surface of a doughnut-shaped structure along its meridians, which can be visualized as a head-to-tail arrangement of magnetic dipoles along a circular path [15,16]. As the third kind of electromagnetic moment, TD resonance has unique electromagnetic characteristics, such as non-radiating mode and confined electric field mode, and their strength depends on the time derivative of the electromagnetic waves [17,18,19]. However, the detection of TD excitations is challenging. The TD is weakly coupled to free space, and TD can be masked by much stronger electromagnetic effects, such as electric and magnetic dipoles, and even electric quadrupoles [20,21,22,23,24,25,26].

Metamaterials (MMs) are broadly defined as artificial, effectively homogeneous electromagnetic structures with unusual properties not readily available in nature. MMs are of great importance to find a way to solve the problems confronted by natural materials. They are usually represented by an array of electric multipoles and magnetic multipoles. By properly designing the structure of metamolecules, the electromagnetic properties can be tailored at will to obtain any multipole radiation pattern. Hence, designing a new structure in order to enhance the TD response, as well as suppressing the electric and/or magnetic multipole resonance is a useful procedure to investigate interesting physical characteristics of TD resonances. MMs provide support to acquire further understanding of the interaction between electric multipole resonances and magnetic multipole resonances in TD resonance.

In our research, we proposed a dual TD MM with a U-shaped modified structure under a terahertz regime, which exhibits low quality factor (Q) and high Q TD resonances at different frequencies simultaneously. This TD MMs had a planar structure instead of a 3D structure, which is easy to fabricate, and had a stable resonance output. Thus, it is a good method to realize the unique characteristics of TD and has potential applications at THz frequencies. We investigated the variation of dual TD resonances by changing the distance between the two coplanar U-shape split ring resonators (USRRs) and the arm width of the resonator in the metamolecule structure by looking into the surface current and magnetic-field distribution. In order to understand the mechanism of TD resonance more in depth, quantitative calculations of scattering powers for the multipoles in the metamaterial were carried out. Utilizing the characteristics of dual TD MMs in THz regime could provide various applications at THz frequencies, such as electromagnetically-induced transparency, nonreciprocal refraction, dichroism, nonlinear optical activity, plasmonic sensors, narrow-band emissions and detectors, and effective cloaking and absorbing materials [27,28,29,30,31,32]. Our work suggests that it is possible to realize any two applications of dual TD MMs in one device under a terahertz domain. It can also facilitate strategies for developing TD resonance on not only freestanding but also on flexible substrates, which can feasibly be used in many applications.

## 2. Sample Design and Characterization

**Figure 1:**
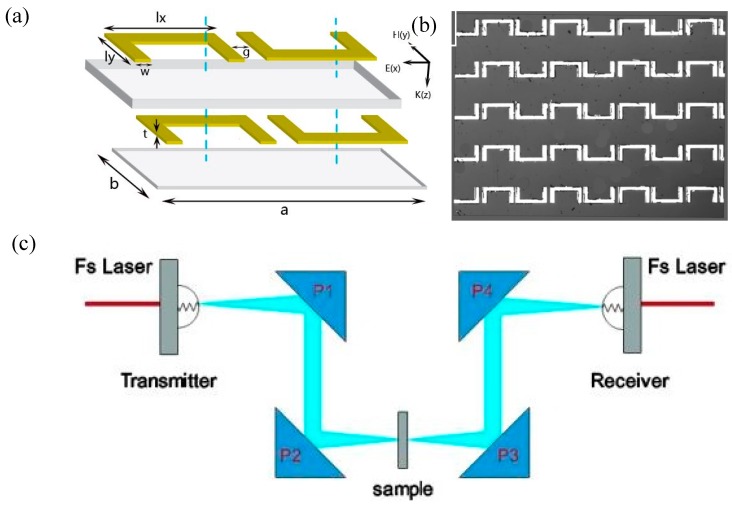
(**a**) Schematic of the proposed MMs (metamaterials) metamolecule. (**b**) Microscope image of the fabricated sample with g = 10 μm. (**c**)THz-TDS system.

The schematic of the proposed MMs is shown in Figure 1a. The proposed structure was simplified from 3D structures to a stack of 2D planar structures, thus the fabrication was simplified. The proposed MMs was composed of two metallic pattern layers; a polyimide spacer layer (20 μm thick) and a polyimide substrate layer (5 μm thick). Each metallic pattern layer had the same metallic pattern, which consisted of two USRRs. The coplanar USRRs had 180° rotational symmetry about the z-axis, in order to form the time-reversal symmetry breaking and space-inversion symmetry breaking. Polyimide film was chosen as the spacer and the substrate, due to its transparency in both THz and visible regimes, thus enabling the aligned photolithography fabrication. The outer dimension of USRRs (lx × ly) was 152 μm × 80 μm and the periodicity of the metamolecule (a × b) was 168 μm × 104 μm. The arm width of the resonator (w) was 18 μm and the distance between the two coplanar USRRs (g) was 10 μm. The resonator was made up of 200-nm-thick (t) aluminum metal.

All the samples were on the XY plane, and the terahertz radiation excited the samples at normal incidence with the electric field linearly polarized along the x-axis (Figure 1a). Since four USRRs were in close proximity, the nearest-neighbor mutual interactions or hybridizations between them played a dominant role in their response to external excitations [27,28]. In order to investigate the mutual electromagnetic effects between the two coplanar USRRs in the same metamolecule, the two coplanar USRRs merged to form a single enlarged double-ring USRRs, the parameter g was varied (g = 20, 0, 10, and −18 μm) and the samples were fabricated accordingly. The w (w = 12, 14, 18, and 26 μm) was varied and affected the mutual effect of the USRRs, then the samples were fabricated accordingly.

The proposed MMs were fabricated using conventional photolithography and metallization processes. Firstly, the liquid polyimide of PI-5878G (HD Micro Systems TM) was spin-coated on a silicon wafer in order to form the 5 μm substrate, which was helpful to simplify the fabrication process. The thickness of the polyimide spacer could be precisely controlled by adjusting the curing temperature and the spin rate. Secondly, the first metal pattern layer was fabricated using conventional photolithography, followed by the deposition of 200 nm aluminum using vacuum coating equipment, then rinsing in acetone for several minutes. Thirdly, the polyimide spacer layer (20 μm) was fabricated on it. Fourthly, the second metal pattern layer was fabricated using the above-mentioned method. Finally, the patterned MMs structures on the polyimide substrate were peeled off from the silicon substrate. One of the microscope images of the sample with g = 10 μm is shown in Figure 1b. The TD MMs were characterized by THz-time domain spectroscopy (TDS) in a broadband, photoconductive switch-based system that consisted of four parabolic mirrors in an 8-F confocal geometry [33,34], as shown in Figure 1c. This confocal geometry based on a parabolic mirror and bare silicon reference, can highly reduce the possible dispersion. The measurements were directly performed with the MMs attached to a well-defined optical aperture at room temperature in a dry air environment to eliminate the absorption of terahertz waves by the water vapor present in the atmosphere.

## 3. Results and Discussion

**Figure 2:**
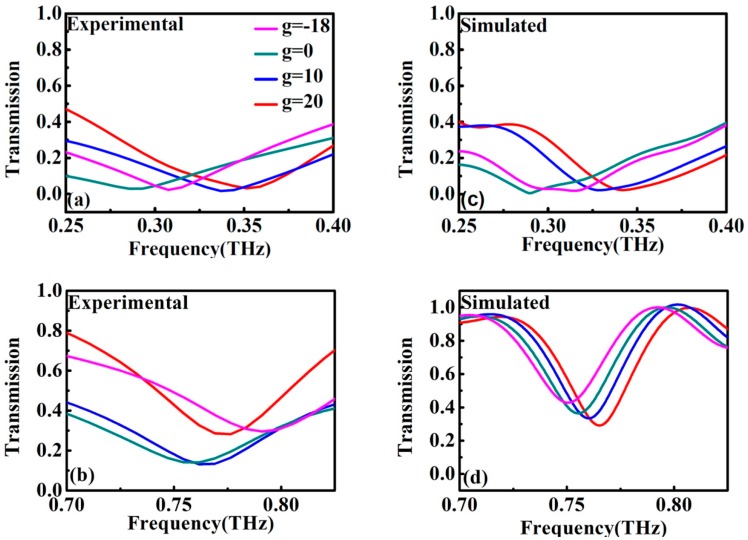
Experimental (**a**,**b**) and simulated (**c**,**d**) amplitude transmission spectra for samples with different values of g, when the E field is parallel to the *x*-axis.

Figure 2a,b show the measured transmission spectra of the proposed MMs samples with different values of g. Two resonances were observed in all samples: the low frequency (LF) resonance at around 0.35 THz, and the high frequency (HF) resonance at around 0.75 THz. As shown in Figure 2a, the LF resonance frequency first decreased then increased as a function of g, that is, the LF resonance frequency shifted to a lower frequency as the g increased from −18 μm to 0 μm, while the LF resonance frequency shifted to a higher frequency as the g further increased to 20 μm. The variation of the HF resonance frequency showed the same tendency as the LF resonance frequency, as shown in Figure 2b. The Q factor was defined as the ratio of resonance frequency to the full width at half maximum. When g = 10 μm, two excitations with the highest Q factor were obtained, and the two excitations were both confirmed as TD resonances, which will be introduced in detail later. For the HF TD resonance at 0.76 THz, the Q (~10.31) was almost six times stronger than that of the LF TD resonance at 0.32 THz (~1.82).

The parameters of w also affected the electromagnetic characteristics of the proposed MMs, which are illustrated in Appendix A. The LF resonance frequency shifted to a higher frequency as the w increased from 12 to 26 μm due to the inductive coupling between the two metallic layers. However, the w had almost no effect on the HF resonance frequency.

For the purpose of understanding the TD characteristics, numerical simulations were performed the in time domain by using the commercially available software CST Microwave Studio. The aluminum metal was described by the Drude model, where the angular frequency dependent permittivity is given by ε(ω) = ε_∞_ − [$\omega_{p}^{2}$/ω(ω + iΓ)], with the plasma frequency ω_p_ set as 22.43 × 10^15^ rads^−1^ and the damping rate 124.34 × 10^12^ rads^−1^ [35], and the dielectric constant of the polyimide is ε = 3.1 × (1 + 0.02i) [36]. As illustrated in Figure 2, the experimental and simulated results were in good agreement except for some acceptable variations, which could be due to the deviation in the thickness of the polyimide arising from the fabrication process.

**Figure 3:**
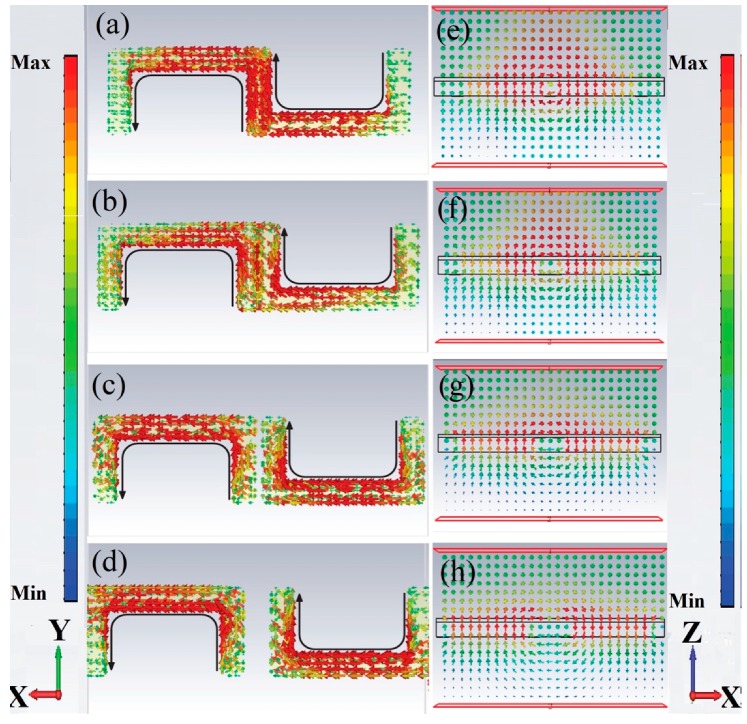
Simulated surface currents (**a**–**d**) and magnetic field (on the XZ plane at Y = 0 (**e**–**h**) of resonator at low frequency resonances.

In order to explore the variation of the LF TD resonance with different g (g = −18, 0, 10, and 20 μm), the surface currents and magnetic field (on the XZ plane at Y = 0) induced in the MMs were investigated, as shown in Figure 3. At g = −18 μm, the current density was concentrated upon the vertical metal strip in the center of the metamolecule, and the induced magnetic dipole formed around the metal strip on the basis of the right hand rule. As the value of g increased to 10 μm, the two coplanar USRRs departed from each other. The induced current formed a loop along the USRR, which is the inductive-capacitive (LC) resonance, then the magnetic dipole type excitation was induced in the metallic resonator [37,38]. Two coplanar USRR resonators had their magnetic dipole type excitation pointing in the upward or downward direction, perpendicular to the MM surface. The neighboring resonators were flipped over in a different manner in one direction, hence their magnetic moment pointed in the opposite direction along the z-axis according to the right hand rule. The TD resonance formed due to the near coupling between the horizontal USRRs and the hybridization effect of trapped modes in the vertical USRRs. As shown in Figure 3e–g, the induced magnetic field was arranged head-to-tail, and the obvious vortex of the magnetic field was observed, which are typical characteristics of TD resonance [12,15]. Thus, LF TD resonance was induced by LC resonance. However, when the g further increased to 20 μm, the current density focused on the horizontal strips of the metamolecule. The mutual effect between the two coplanar USRRs was too weak to form the TD resonance, and TD resonance was hardly observed, as shown in Figure 3h.

**Figure 4:**
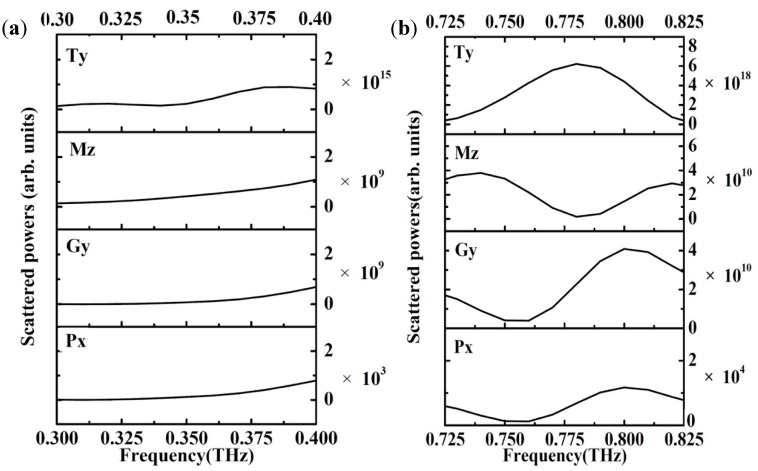
Decomposed scattering powers corresponding to different multipole moments (g = 10 μm). (**a**) around LF resonance (**b**) around HF resonance.

**Figure 5:**
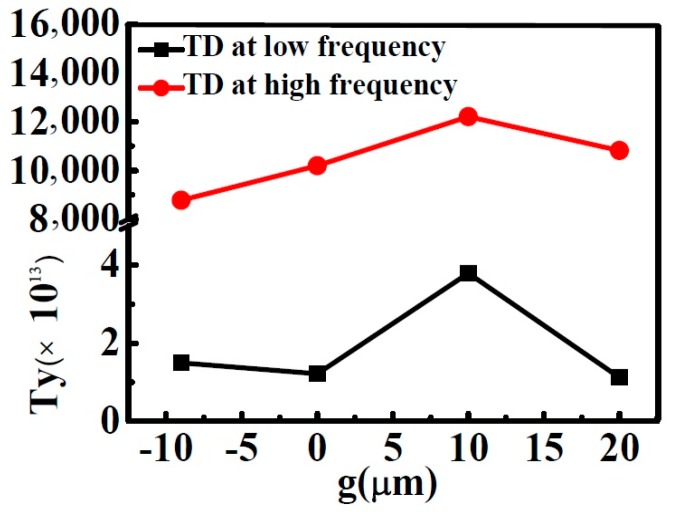
Scattering power of Ty as a function of g.

To further analyze the TD resonance in the MMs, we carried out a quantitative calculation regarding the scattering powers of various multipoles by using the multipole scattering theory according to the volume current density distribution (for the calculation method see Appendix B) [11,12,24]. Four USRRs corresponded to a mixture of magnetic multipoles, electric multipoles, and toroidal multipoles. In order to simplify the analysis, all higher multipoles were ignored. At THz normal incidence, the electric dipole vector was along the x-axis, the magnetic dipole vector (Mz) was parallel to the z-axis, the TD vector (Ty) was parallel to the y-axis, and the electric circular dipole (Gy) was along the z-axis. Figure 4 illustrates the calculated scattering powers of Ty, Px, Mz, and Gy, when g = 10 μm. As shown in Figure 4a, Ty was much stronger than any other multipoles, and made the strongest contribution to a resonant dip at 0.32 THz. We further investigated the relationship between Ty and g by plotting the scattering powers of Ty as a function of g in Figure 5. The relationship between the scattering powers of Ty and g is nontrivial: when g increased from −18 to 10 μm, the strength of the LF TD resonance was enhanced. When g further increased to g = 20 μm, the strength of the LF TD resonance was suppressed. When g = 10 μm, the LF TD resonance had the strongest Ty. The calculated results coincided with the numerical simulation results shown in Figure 3.

**Figure 6:**
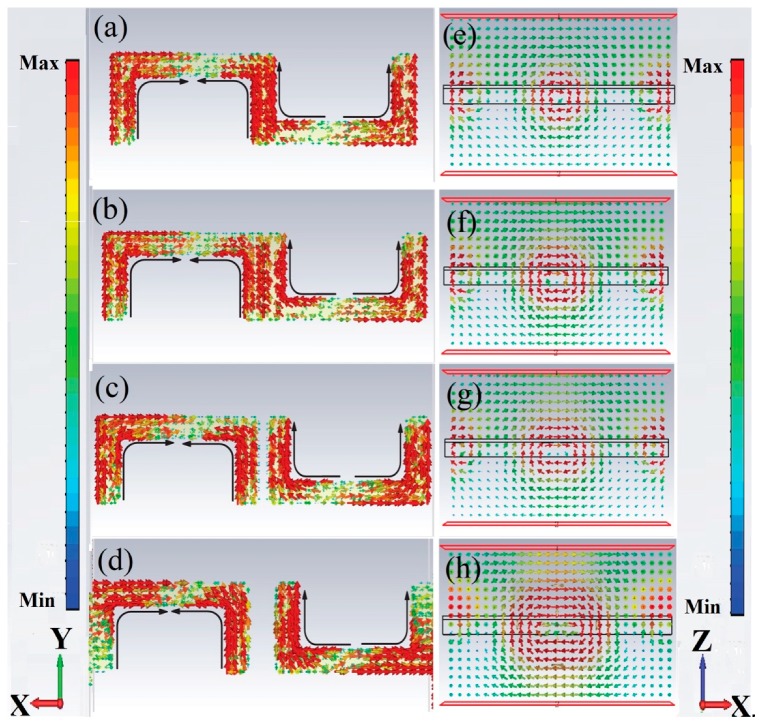
Simulated surface currents (**a**–**d**) and magnetic field (on XZ plane at Y = 0 (**e**–**h**)) of resonator at high frequency resonances.

In our research, the HF resonance was also demonstrated as TD resonance. In order to investigate the mechanism of the TD resonance more in depth, the simulated surface currents and magnetic field at HF resonances were discussed. As shown in Figure 6a–d, the currents flow opposite along the strips in the same USRR, which is the typical dipole resonance of split ring resonators [37,38]. Focusing on the strips in the center of the metamolecule, the currents flowing in the adjacent vertical metal strips were anti-phase, and the magnetic dipole was formed oppositely. The head-to-tail magnetic dipoles were tightly confined around the metal bar in a smaller circular region. A strong antiparallel surface current distribution in neighboring USRRs pronounced a relatively stronger coupling in TD resonance. Thus, it could be confirmed that the HF TD resonance was induced by dipole resonance. The smaller TD around the edge of the metamolecule was observed to be due to the margin effect of the metamolecule. As shown in Figure 4b, the contribution of the Mz was strongly suppressed, while Ty had the strongest resonance at 0.76 THz. The relationship between the scattering power of Ty and g around 0.76 THz was the same as the relationship between the Ty and g around 0.32 THz, as shown in Figure 5. When g = 10 μm, the scattering powers of Ty was the strongest for the HF TD resonance.

The magnetic dipole resonance of a single USRRs was split into two discrete resonances that originate from the hybridization of magnetic resonance in the stacked USRRs. The hybridization effect was mainly attributed to mutual inductive and capacitive coupling between the USRRs, and the two resonances were far from the original magnetic mode of USRRs due to extreme overlap and close proximity. In previous works, the energy level of the degenerated resonance modes was split into toroidal and magnetic resonances, and the energy level was determined by the electric dipole interaction [24,25,26]. In our work, the energy level of the degenerated resonance modes was split into LC-induced TD resonance and dipole-induced TD resonance at two different frequencies when g ≤ 10 μm. The energy level of the dipole resonance was much higher than the LC resonance in the typical split ring resonators. The electric multipole interaction played an important role in determining the energy level of the resonant modes. The strength depends on the time derivative of electromagnetic waves, hence the interaction energy of the TD resonances also increased with the shift from lower to higher frequencies. Compared with the LF TD resonance, the HF TD resonance showed a larger field confinement per unit volume, attributed to the tightly confined time varying magnetic field in a smaller circular region. The coupling between the four USRRs was enhanced, and the TD was weakly coupled to the free space, and the radiative losses were reduced, thus the Q factor was dramatically enhanced. In our research, dual TD excitations were experimentally demonstrated at the LF and HF frequencies simultaneously by proper adjustment of the g value. The Q factor of the HF TD resonance was much higher than that of the LF TD resonance in the proposed MMs. The nature of the high Q factor in the TD response was helpful to make efficient resonant MMs devices.

## 4. Conclusions

We experimentally demonstrated planar freestanding dual TD MMs with normal incident light. The structure of the metamolecule was designed precisely in order to obtain time-reversal symmetry breaking and space-inversion symmetry breaking, which is vital to the formation of TD resonance. By adjusting the g value, both low Q and high Q TD resonances were obtained simultaneously at two different frequencies in the MMs. With the interaction of the USRRs, the energy levels of the USRRs were split into two resonances, corresponding to the LC-induced TD resonance at the LF frequency and dipole-induced TD resonance at the HF frequency, respectively. In HF TD resonance, the head-to-tail magnetic dipole was confined to a small circular region, which led to increased Q. The Q of the HF TD resonance was found to be much higher than that of the LF TD resonance. The dual TD MMs on the freestanding substrate could have enormous potential applications in terahertz functional metadevices.

## Figures and Tables

**Figure A1:**
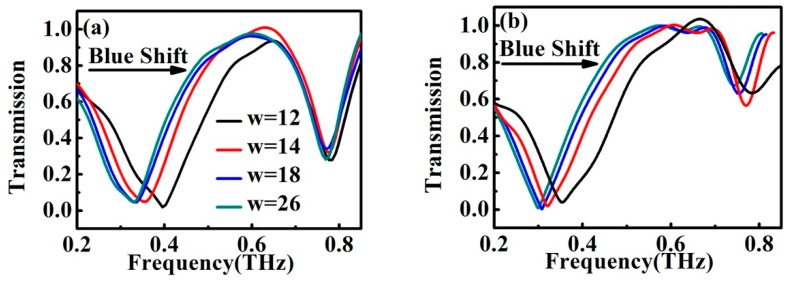
Experimental (**a**) and simulated (**b**) amplitude transmission spectra for samples with different value of w, when the E field is parallel to x-axis.

## Appendix A

Figure A1a,b shows the measured and simulated transmittance of MMs samples with different w. The frequency of the LF TD resonance shifted to a higher frequency as the w increased from 12 μm to 26 μm. The blue shift in toroidal resonance indicates a weaker coupling between the USRRs. As the value of w increased to 26 μm, the interaction between the two USRRs weakened. However, the w had little influence on the HF TD resonance. The LF TD frequency can be adjusted by the w, while having almost no effect on the HF TD frequency.

## Appendix B

Dynamic toroidal multipoles constitute an independent family of elementary electromagnetic sources. The lowest order toroidal multipole, the toroidal dipole, corresponds to currents flowing on the surface of the torus, along its meridians. The moment of the electric dipole, magnetic dipole, electric circular dipole, and toroidal dipole directed along the axis of torus is given by the following equation [11,12,24]:

Electric dipole: $P=\frac{1}{i\omega }\int jd^{3}r$
Magnetic dipole: $M=\frac{1}{2c}\int \left(r\times j\right)d^{3}r$
Electric circular dipole: $G=\frac{1}{2}\int \left(r\times p\right)d^{3}r$
Toroidal dipole: $T=\frac{1}{10c}\int \left[\left(r\times j\right)r-2r^{2}j\right]d^{3}r$

where *r* is the coordinate vector with its origin placed at the center of torus, *c* is the speed of light, and *j* is the current density. The simulated current density (*j*) was obtained via CST Microwave Studio simulations, which are used to calculate the strength of toroidal dipole by means of MATLAB programs.
